# In Vitro Characterization of Aerosolized Albuterol Generated by a Jet Nebulizer and Delivered through a Heated Flow Nasal Cannula System

**DOI:** 10.3390/ph15101281

**Published:** 2022-10-18

**Authors:** Ariel Berlinski, Joshua Spiva

**Affiliations:** 1 Division of Pediatric Pulmonary and Sleep Medicine, Department of Pediatrics, College of Medicine, University of Arkansas for Medical Sciences, Little Rock, AR 72202, USA; 2 Pediatric Aerosol Research Laboratory, Arkansas Children’s Research Institute, Little Rock, AR 72202, USA; 3 Arkansas Children’s Research Institute, Little Rock, AR 72202, USA

**Keywords:** jet nebulizer, particle size, aerosol MMAD, heated flow cannula, cascade impactor, pediatrics, drug delivery

## Abstract

Pediatric patients receiving respiratory support with heated flow nasal cannula (HFNC) systems frequently receive inhaled medications. Most available data have been obtained with vibrating mesh nebulizers that are expensive. Data are lacking regarding the feasibility of using less expensive devices such as continuous output jet nebulizers. The characteristics of the aerosols generated by jet nebulizers operated at different conditions (6 and 9 L/min) were studied alone and connected to a HFNC system and different size cannulas using a cascade impactor and spectrophotometry (276 nm). Aerosol characteristics changed while traveling through the HFNC system. Initial size selection occurred at the exit of the circuit (before connecting to the cannula) with all aerosol <5 µm. Nasal cannula size further selected aerosols and reduced drug delivery. The operating flow of the nebulizer did not affect the delivered mass but higher flows generated smaller particle size aerosols. The addition of supplemental flow significantly reduced the delivered mass. The measured aerosol characteristics would likely result in intrapulmonary deposition. The delivery of aerosolized albuterol generated by a continuous output nebulizer placed in the inlet of a HFNC system and connected to large or XXL cannulas is feasible.

## 1. Introduction

Heated flow nasal cannula (HFNC) systems are widely used in pediatrics to provide respiratory support [1]. Many of these patients also need inhaled medications and practitioners use inline concomitant delivery [2]. A recent survey revealed that 75% of practitioners reported giving aerosolized medications to children receiving HFNC support [3]. Therefore, it is clinically relevant to understand the fate of the aerosols generated by nebulizers connected to HFNC systems.

Several in vitro studies have evaluated the characteristics of aerosols generated by vibrating mesh nebulizers and released by nasal cannulas of pediatric size after travelling through the HFNC system [4,5,6,7,8]. These devices are commonly placed in the inlet of the humidifier. These types of nebulizers have the advantage of not needing flow to generate the aerosols, thus letting the flow of the HFNC transport the aerosol [9]. However, these devices are expensive and might not be available in locations with limited resources. Continuous output jet nebulizers use a gas source to generate the aerosol, thus adding a fix flow to the HFNC system. However, they are inexpensive and widely available. A previous in vitro pediatric study reported an increase in drug delivery when low flows were used in a HFNC system with a vibrating mesh nebulizer placed at the inlet of the humidifier. [10] We found one report of mass median aerodynamic diameter (MMAD) (1.07 µm) of a jet nebulizer placed at the inlet of the humidifier, operated at one flow, and using a neonatal nasal cannula [7]. Another study from the same group evaluated aerosol MMAD and mass delivered by a jet nebulizer inline a HFNC system in an adult model [11]. Their model used flows between 30 and 60 L/min and the nebulizer was placed in different positions. They reported an MMAD of 1.40 µm.

Aerosols with MMAD < 5 µm are likely to be deposited in the lungs, therefore, the particle size characterization of aerosols released by nasal cannulas connected to HFNC is relevant [12].

The aim of the study was to understand the fate of aerosols generated by a continuous output jet nebulizer as they travel through a HFNC system and are released through nasal cannulas. Previous in vitro and in vivo studies have reported the feasibility of the drug delivery of aerosols via HFNC using vibrating mesh nebulizers [2,10]. We planned to determine the feasibility of drug delivery through these systems by evaluating the delivered mass and aerosol characteristics. We hypothesized that particle size and the amount of delivered drug will decrease while the aerosol travels through the HFNC system.

## 2. Results and Discussion

Results are expressed as mean ± SD unless otherwise stated and are summarized in Table 1 and Table 2.

The measurement conditions remained stable (mean, 99%CI; 22.7, 22.6–22.8 °C; and 69.5, 68.4–70.5% for temperature and humidity, respectively). The amount of albuterol remaining in the nebulizer (mean, 99%CI) was 1494 µg, 1464–1525 µg. The solution output (mean, 99%CI) was 1.37 mL, 1.33–1.40 mL. Both the laboratory conditions and nebulizer output were similar across all of the performed studies. This provides confidence that any found differences are likely to be real.

Table 1 includes the outcome measures for nebulizers with Tee-piece operated at 6 and 9 L/min alone, connected to a Y-piece alone, connected to a Y-piece that was connected to the humidifier and circuit, and connected to a Y-piece that was connected to the humidifier, circuit, and small, large, and XXL cannulas.

Table 2 includes the outcome measures for nebulizers with Tee-piece operated at 6 and 9 L/min (with/without additional flow through the HFNC) connected to a Y-piece that was connected to the humidifier, circuit, and large and XXL cannulas.

### 2.1. MMAD

All aerosols had an MMAD suitable for intrapulmonary deposition. When the nebulizer was operated at 6 L/min, a progressive decrease in MMAD was noticed as more parts were added to the delivery system (*p* < 0.0001) (Table 1). The addition of the humidifier and the circuit reduced the MMAD by 25%. The addition of the cannula further reduced MMAD by another 10% and 22% for XXL and large cannulas, respectively, highlighting the effect of cannula size on aerosol delivery. When the nebulizer was operated at 9 L/min, a progressive decrease in MMAD was noticed as more parts were added to the delivery system (*p* < 0.0001) (Table 1). The addition of the humidifier and the circuit reduced the MMAD by 39%. The addition of the cannula further reduced the MMAD by 4% and 13% for the XXL and large cannulas, respectively. These data are consistent with Bhashyam et al., who reported a progressive decrease in particle size as more parts were added to the system [4]. They also found a direct relationship between the cannula size and particle size. Their study had a different methodology including the use of laser diffraction for particle size characterization, the use of a vibrating mesh nebulizer, and the use of low flows (3 L/min).

**Figure 1:**
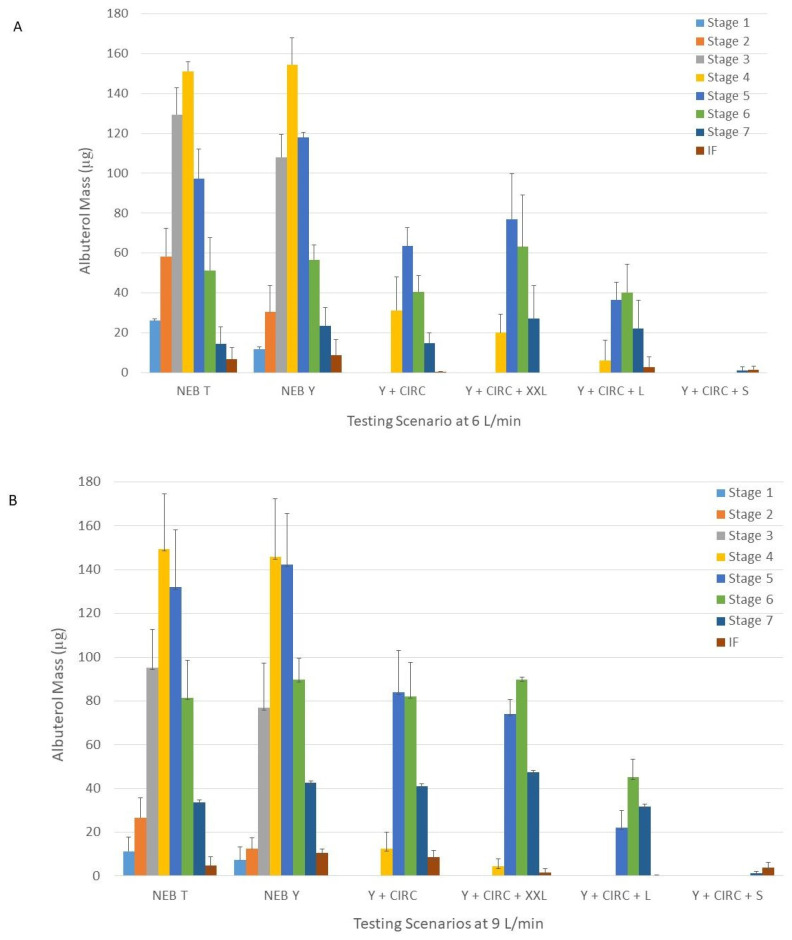
Drug distribution across the different stages of the Next Generation Impactor with nebulizers with the Tee-piece, with the Tee-piece connected to the Y-piece, with the Tee-piece connected to the Y-piece connected to the humidifier and circuit, and with the Tee-piece connected to the Y-piece connected to the humidifier, circuit, and small, large, and XXL cannulas and operated at 6 (panel (**A**)) and 9 L/min (panel (**B**)), respectively. Panel A = 6 L/min. Panel B = 9 L/min. NEB T = nebulizer with the Tee-piece. NEB Y = nebulizer with the Tee-piece connected to the Y-piece. Y + CIRC = nebulizer with the Tee-piece connected to Y-piece connected to the humidifier and circuit. Y + CIRC + XXL = nebulizer with the Tee-piece connected to the Y-piece connected to the humidifier, circuit, and XXL cannula. Y + CIRC + L = nebulizer with the Tee-piece connected to the Y-piece connected to the humidifier, circuit, and large cannula. Y + CIRC + S = nebulizer with the Tee-piece connected to the Y-piece connected to the humidifier, circuit, and small cannula.

The shift in aerosol size that occurred with both operating flows as the setup becomes more complex can be better appreciated in Figure 1. The captured mass became skewed toward final stages of the Next Generation Impactor (NGI model 170, MSP Corporation, Shoreview, MN, USA), in contrast to a more symmetric disposition seen with the nebulizer alone. This pattern reflects the retention by the impaction of large sized particles before the aerosol is released by the cannula. The mayor reduction occurs once the aerosols leave the circuit.

**Figure 2:**
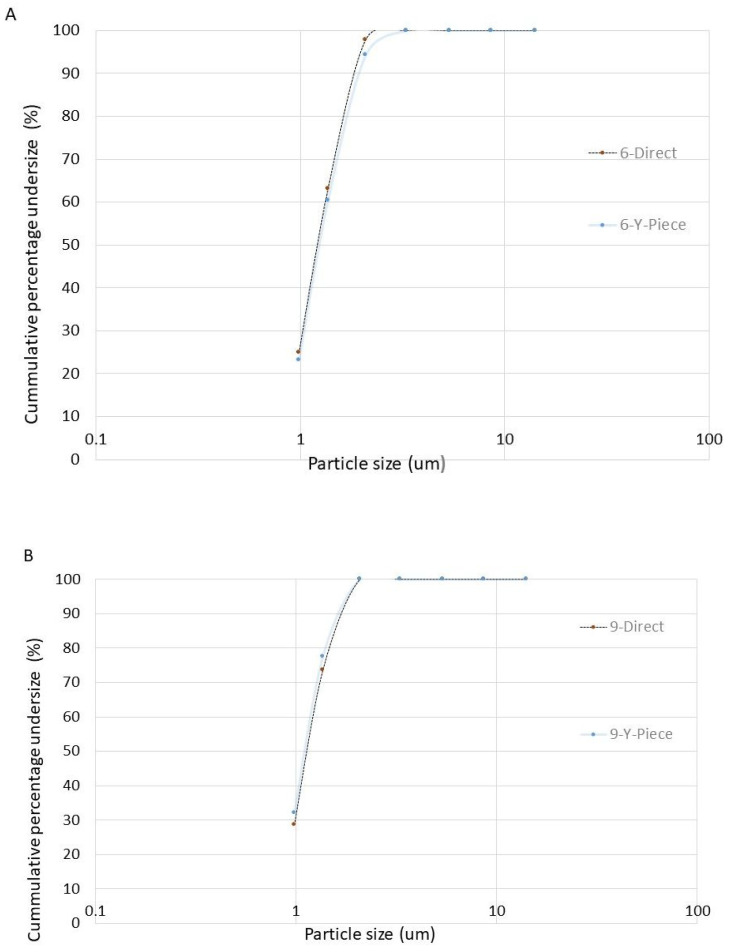
Plot of the logarithmic particle size against the cumulative percentage undersize for aerosols released by a large cannula when the nebulizer with Tee-piece was operated at 6 L/min (panel (**A**)) and 9 L/min (panel (**B**)) and connected to the humidifier directly or via Y-piece. Panel A = 6 L/min. Panel B = 9 L/min. 6-Direct = nebulizer operated at 6 L/min with the Tee-piece attached directly to the humidifier connected to circuit and large cannula. 6-Y-piece = nebulizer operated at 6 L/min with the Tee-piece attached to a Y-piece connected to the humidifier connected to circuit and large cannula. 9-Direct = nebulizer operated at 9 L/min with the Tee-piece attached directly to the humidifier connected to the circuit and large cannula. 9-Y-piece = nebulizer operated at 9 L/min with the Tee-piece attached to a Y-piece connected to the humidifier connected to circuit and large cannula.

The MMAD of aerosols released by a large cannula were similar when the nebulizer with the Tee-piece was connected to the humidifier directly or via the Y-piece (*p* = 0.46 and *p* = 0.19 for 6 and 9 L/min, respectively) (Figure 2).

Pairwise comparisons of MMAD showed that when the nebulizer was operated at 9 L/min, the size of the aerosols was smaller than when the nebulizer was operated at 6 L/min (*p* < 0.024).

The MMAD in our study was larger than in Reminiac et al. when using a jet nebulizer (1.4 µ) [11]. This could be explained in part because they operated the HFNC at a larger flow (30 L/min). The MMAD in our study was smaller than Watts et al. (2.3 µ), who used a vibrating mesh nebulizer, an infant cannula, and a similar HFNC flow (5 L/min) [6]. Methodological differences including the use of concomitant simulated breathing, and the type of nebulizer could in part explain the differences. Reminiac et al., in another study, reported an MMAD of 1.07 µm using a jet nebulizer operated at 6 L/min with an additional flow of 2 L/min by HFNC using a neonatal size cannula [7]. The total flow exceeded the maximum flow recommended by the manufacturer. This could have resulted in larger deposition within the system.

**Figure 3:**
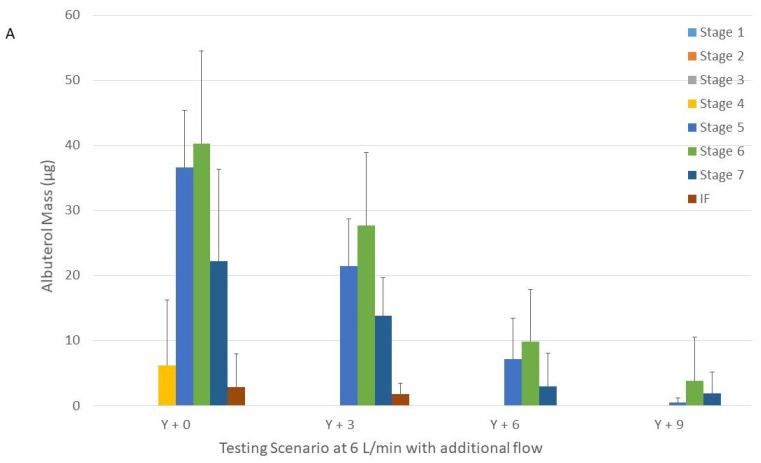

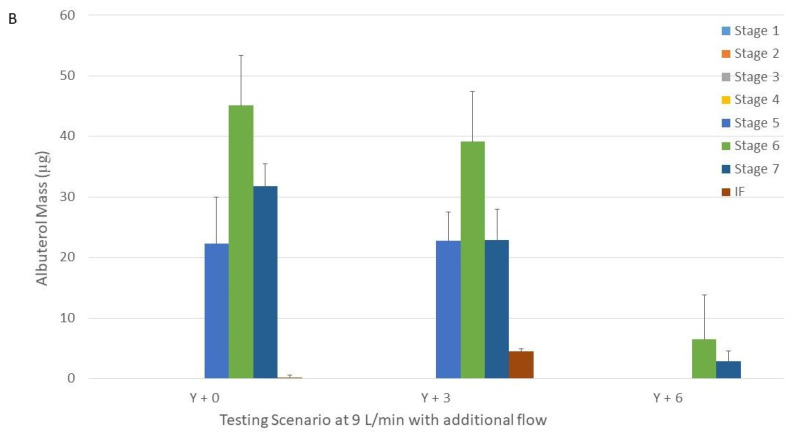
Drug distribution among the different stages of the Next Generation Impactor with nebulizers with Tee-piece operated at 6 (panel (**A**)) and 9 L/min (panel (**B**)) connected to a Y-piece connected that was to the humidifier, circuit, and large cannula with and without additional flow. Panel A = 6 L/min. Panel B = 9 L/min. Y + 0 = nebulizer with Tee-piece connected to a Y-piece that was connected to the humidifier, circuit and large cannula without additional flow. Y + 3, Y + 6, and Y + 9 were similar to Y + 0, except that they had 3, 6, and 9 L/min of additional flow, respectively provided through the Y-piece.

The addition of 3 L/min to the nebulizer operated at 6 L/min did not change the MMAD for the large and XXL cannulas (*p* = 0.20 and *p* = 0.56, respectively) (Table 2). We were unable to calculate the MMAD when 6 and 9 L/min were added and the large cannula was used due to the skewed and low amount of drug recovered in the NGI (only in 3 NGI stages) (Figure 3).

The addition of 3 and 6 L/min to the nebulizer operated at 9 L/min did not change the MMAD for the large and XXL cannulas (*p* = 0.43 and *p* = 0.08, respectively) (Table 2). We were unable to calculate the MMAD when 6 L/min was added and the large cannula was used due to the skewed and low amount of drug recovered in the NGI (only in 2 NGI stages) (Figure 3).

Operating the nebulizer at higher flows (9 vs. 6 L/min) seemed to improve drug delivery. Overall, the resulting MMAD is expected to provide central and peripheral lung deposition [12,13].

### 2.2. GSD

The GSD of the nebulizer connected to the Tee-piece and connected to the Y-piece were similar (*p* = 0.98 and *p* = 0.69 for 6 and 9 L/min, respectively) (Table 1). All the other aerosols were less heterodisperse (*p* < 0.0001). This behavior was expected based on the progressive size selection that occurred as the setup became more complex (Figure 1).

Pairwise comparisons of GSD showed that when the nebulizer was operated at 9 L/min, the aerosols were less heterodisperse than when operated at 6 L/min only when the cannulas were connected (*p* < 0.04) (Table 1).

The GSD of aerosols released by a large cannula were similar when the nebulizer with a Tee-piece was connected to the humidifier directly or via the Y-piece (*p* = 0.47 and *p* = 0.13 for 6 and 9 L/min, respectively).

The addition of supplemental flow to the nebulizer operated at either 6 or 9 L/min with either a large or a XXL did not change GSD (*p* > 0.09).

The GSD reported in our study was similar to that of Reminiac et al. (1.5) [11]. Other studies did not report the GSD data.

### 2.3. FPF

FPF is considered as the fraction of aerosol that will most likely result in intrapulmonary deposition. All aerosols released by the nasal cannulas were likely to be deposited in the lungs. The addition of the Y-piece resulted in a 10 and 17% increase in FPF for the nebulizer operated at 6 and 9 L/min, respectively (Table 1). However, the aerosols released by the circuit and the cannulas had similar FPF around 100% (*p* = 0.99 and *p* = 0.99 for 6 and 9 L/min, respectively). This behavior can be once again explained by the size selection that occurred via impaction as the aerosols traveled through the HFNC system. All the aerosols released by cannulas are suitable for intrapulmonary deposition. These results are similar to those reported by Reminiac et al. using higher flows through the HFNC system [11].

The FPF of aerosols released by a large cannula were similar when the nebulizer with a Tee-piece was connected to the humidifier directly or via the Y-piece (*p* = 0.42 and *p* = 0.42 for 6 and 9 L/min, respectively).

Pairwise comparisons of FPF showed that when the nebulizer was operated at 9 L/min, the aerosols had higher FPF than when operated at 6 L/min only when the nebulizers were operated alone (Table 1). However, once the humidifier and circuit were added to the setup, the FPF became similar (*p* > 0.05).

The addition of supplemental flow to the nebulizer operated at either 6 or 9 L/min with either a large or a XXL did not change the FPF (*p* > 0.09) (Table 2).

### 2.4. MASS

The MASS of the nebulizer with Tee-piece alone or connected to the Y-piece was similar (*p* = 0.97 and *p* = 0.99 for 6 and 9 L/min, respectively) and larger than in any other setups (*p* < 0.0001) (Table 1). The MASS released by large and XXL cannulas averaged 8.1 and 4.2%, respectively. The MASS was lower for the small cannula that for any other experimental setups and was almost negligible (Table 1). This is consistent with previous studies by Bashyam et al., Perry et al., and Bennett et al., who studied vibrating mesh nebulizers and reported an increase in drug delivery with an increasing cannula size [4,5,8].

Pairwise comparisons of MASS showed no differences between the nebulizer operated at 6 and 9 L/min (*p* > 0.08) (Table 1). These findings are different from Reminiac et al., who found an inverse relationship between the flows and delivered mass [11]. However, these differences can be explained by the fact that they studied flows of 30, 45, and 60 L/min while we used 6 and 9 L/min.

The addition of supplemental flow to the nebulizer operated at 6 L/min when a large cannula was used resulted in a decrease in MASS when 9 L/min (*p* = 0.01), but not when 3 and 6 L/min were added (*p* = 0.54 and *p* = 0.051, respectively) (Table 2). The addition of supplemental flow to the nebulizer operated at 6 L/min when a XXL cannula was used resulted in a decrease in MASS when 6 and 9 L/min (*p* = 0.007 and *p* = 0.003, respectively), but not when 3 L/min were added (*p* = 0.16) (Table 2).

The addition of supplemental flow to the nebulizer operated at 9 L/min when a large cannula was used resulted in a decrease in MASS when 6 L/min (*p* = 0.007), but not when 3 L/min were added (*p* = 0.70) (Table 2). The addition of supplemental flow to the nebulizer operated at 9 L/min when a XXL cannula was used resulted in a decrease in MASS when 3 and 6 L/min (*p* = 0.0005 and *p* = 0.0001, respectively) (Table 2). These findings are similar to Reminiac et al. and Perry et al., who reported an inverse relationship between the flows and delivered mass [5,11].

The MASS of aerosols released by a large cannula was similar when the nebulizer with a Tee-piece was connected to the humidifier directly or via a Y-piece (*p* = 0.36 and *p* = 0.37 for 6 and 9 L/min, respectively).

### 2.5. Special Experimental Setups

We compared the characteristics of the aerosols generated by the nebulizer with Tee-piece operated at 6 and 9 L/min with the device placed at the inlet of the humidifier with the Y-piece, and then connected to the circuit and a similar setup but adding the large cannula connector after having removed the cannula. We found similar MMAD, GSD, FPF, and MASS (*p* = 0.58, 0.99, 0.99, and 0.75, respectively) when the nebulizer was operated at 6 L/min. We found similar MMAD, GSD, FPF, and MASS (*p* = 0.99, 0.99, 0.99, and 0.98, respectively) when the nebulizer was operated at 9 L/min. These findings demonstrate that the addition of the connector did not significantly modify the particle size characteristics. However, the full cannula produced a significant reduction in the MMAD (13 and 16% for 6 and 9 L/min, respectively) and MASS (47 and 62% for 6 and 9 L/min, respectively).

We compared the characteristics of the aerosols generated by the nebulizer with Tee-piece placed at the inlet of the humidifier with the Y-piece, and then connected to the circuit and a large cannula or right before a large cannula with the nebulizer operated at 6 L/min with 3 L/min of additional flow. Proximal placement of the nebulizer resulted in a 96% reduction in albuterol MASS with the drug only found in stage 7 and internal filter of the NGI. This finding could be explained by the fact that the aerosol suffered a significant size reduction as it travelled through the circuit, thus decreasing the probability of impaction. Conversely, the aerosol generated before the cannula was more heterogeneous and the larger particles resulted in significant local impaction. In addition, the placement was not convenient for patient use because of possible spills of the medication due to patient movement. Moreover, the introduction of cold dry air into the circuit will produce a reduction in temperature and absolute humidity, potentially increasing the risk of bronchospasm. These findings are consistent with previous findings by Watts el al. and Reminiac et al., who reported a reduction in delivered mass when the nebulizer was moved from the inlet of the humidifier to right before the cannula [6,11]. While Watts used a vibrating mesh nebulizer at low HFNC flows (5 L/min), Reminiac used a jet nebulizer with high HFNC flows (30, 45, and 60 L/min). Bennett et al. reported similar results using a vibrating mesh nebulizer [8].

### 2.6. Cannula

The amount of albuterol captured in large cannulas was similar among the different configurations (*p* = 0.41 and *p* = 0.69 for 6 and 9 L/min, respectively). The amount of albuterol captured in the XXL cannulas was similar among the different configurations (*p* = 0.47 and *p* = 0.68 for 6 and 9 L/min, respectively).

The amount of albuterol deposited in the cannula correlated to the cannula size. Pooled data (median, mean, 99%CI) were 0.5%, 0.5%, 0.3–0.7% for the XXL cannula; 7.6%, 7.7%, 5.9–9.6% for the large cannula; and 8.3%, 9.1%, 4.2–14% for the small cannula. These data showed that smaller size cannulas resulted in larger amounts of drug being retained in them when compared to the larger size cannulas. These data differ from Bhashyam et al., who reported an increasing amount of radiolabeled aerosols with increasing cannula sizes [4]. These differences could be attributed to methodological differences since they used a low flow (3 L/min), vibrating mesh nebulizers, and did not perform biochemical determination of the drug.

We also evaluated the presence of large droplets released by the cannula by evaluating the presence of the drug in the induction port of the NGI. We only found the drug when using the small cannula with the nebulizer operated at 6 L/min (0.15% of loading dose).

### 2.7. Clinical Implications and Study Limitations

Our results suggest that jet nebulizers operated at 6–9 L/min and placed in the inlet of HFNC could be used to deliver bronchodilators when coupled to large and XXL cannulas. Adding extra flow is not recommended. Further studies should be conducted in pediatric patients. Additionally, some of the inefficiency of the system can be overcome by increasing the loading dose. The small size cannula should not be used given its low efficiency. However small size cannulas are recommended for patients lighter than 3.5 kg and younger than 42.5 weeks where the need for nebulized albuterol is not so critical.

This study has several limitations. First, it is an in vitro study without inherent biological variability. However, it used well-accepted methodologies. Second, we used only one brand of nebulizer and we did not study low flows. Finally, we did not utilize simulated breathing, thus overestimating drug delivery.

## 3. Materials and Methods

The experiments were carried out at the Pediatric Aerosol Research Laboratory at Arkansas Children’s Research Institute, Little Rock, Arkansas, 72202, USA.

Three different units of a continuous output jet nebulizer (Hudson Updraft II, Hudson RCI, Temecula, CA, USA) were tested. The manufacturer recommends utilizing flows between 5 and 9 L/min. The nebulizer was connected to the inlet of a heater humidifier (Fisher & Paykel MR850, Fisher & Paykel Healthcare Limited, Auckland, New Zealand) through a spring-loaded Tee-piece adapter capped on one end (Airlife, Vyaire Medical, Mettawa, IL, USA) that was connected to a Y-piece that allowed for a separate supply of additional flow. The humidifier was connected to a heated-wired circuit (RT 330, Fisher & Paykel Healthcare Limited, Auckland, New Zealand) and nasal cannulas designed for pediatric patients (Optiflow Junior 2, Fisher & Paykel Healthcare Limited, Auckland, New Zealand) in sizes small, large, and XXL. The flow ranges recommended by the manufacturer are 0.5–9, 0.5–23, and 1–36 L/min for the small, large, and XXL cannulas, respectively. The patient weight ranges suggested by the manufacturer are 1–3.5, 3.5–18, and >8 kg for the small, large, and XXL cannulas, respectively. The patient age ranges suggested by the manufacturer are 27–42.5 weeks and 37.5 weeks–5.6 years old for the small and large cannulas, respectively. No specific age recommendation is provided for the XXL cannula, but is recommended for those who need a larger size than XL (7–25 kg and up to 12 years old). The cannula should occupy 50% of the naris, thus this parameter combined with age and weight recommendations and required flow can be used to determine the cannula size to be used for a specific patient. The cannula connects to the circuit through an adapter that has two orifices that connect to the cannula tubing. The orifices are in a straight path to the aerosol coming from the circuit in the small and large cannulas, and in the Y-shaped position in the XXL cannula. The diameter of the orifices was 3.3, 5.3, and 11 mm for the small, large, and XXL cannulas, respectively.

### 3.1. Common Procedure

**Figure 4:**
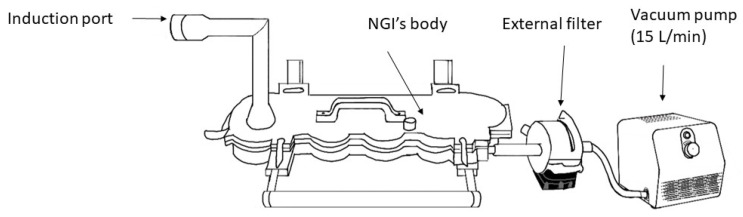
Next Generation Impactor (NGI).

Particle size distribution and mass were measured using the NGI assembled with an internal and external filter and operated at 15 L/min (Figure 4) [14]. The cut-off size for stages 1, 2, 3, 4, 5, 6, and 7 were 14.1 µm, 8.61 µm, 5.39 µm, 3.3 µm, 2.08 µm, 1.36 µm, 0.98 µm, respectively, when the impactor was operated at 15 L/min [15]. Gas flow into the NGI and output from the flowmeters were calibrated with a mass flow meter (Mass Flowmeter 4043, TSI, Shoreview, MN, USA) before each procedure. The NGI was cooled for 60 min at 4 °C prior to use and flow into NGI was recalibrated before starting a new test. The humidity and temperature were monitored prior to each experiment with a thermo-hygrometer (Fisherbrand, Fisher Scientific, Hampton, NH, USA). The albuterol solution 2.5 mg/3 mL (Nephron Pharmaceuticals Co., Orlando, FL, USA) was placed in a dry nebulizer. The devices were weighed dry, after loading the solution, after nebulization was complete, and after adding 10 mL of double-distilled water (APX-60, Denver Instrument, Arvada, CO, USA). Nebulizers were operated with 50 psi air through a back compensated flowmeter 0–15 L/min (Timeter, Allied Healthcare, St. Louis, MO, USA) at 6 and 9 L/min for 5 min. The NGI induction port (IP), cups, and filters, nebulizers and cannulas were eluted with 10 mL of double-distilled water. The eluted drug was analyzed via spectrophotometer (ThermoScientific BioMate 160, Thermo Fisher Scientific, Waltham, MA, USA) at 276 nm. The solution output (mL) and the amount of albuterol remaining in the nebulizer cup was measured to ensure that any of the differences could not be attributed to the solution output or albuterol mass released from the nebulizer.

### 3.2. Experimental Configurations

**Figure 5:**
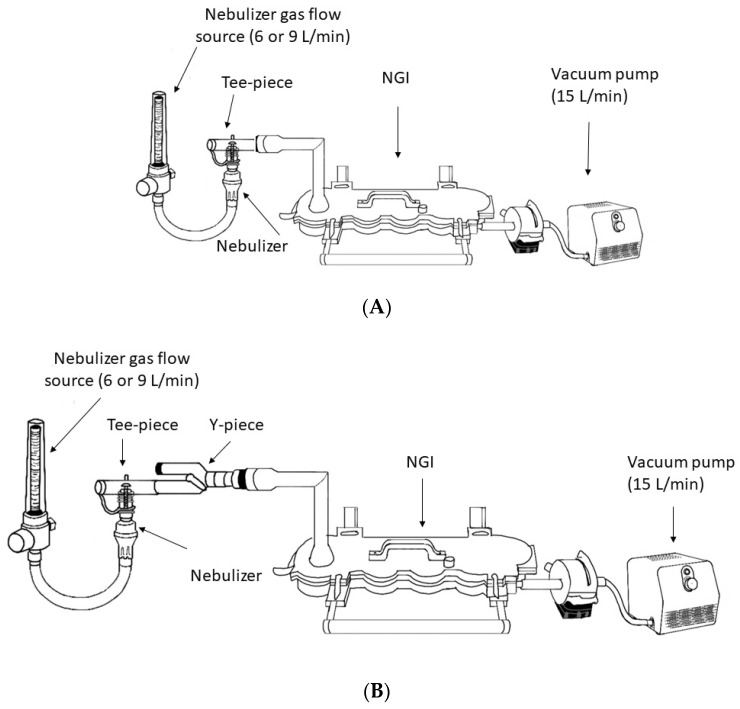

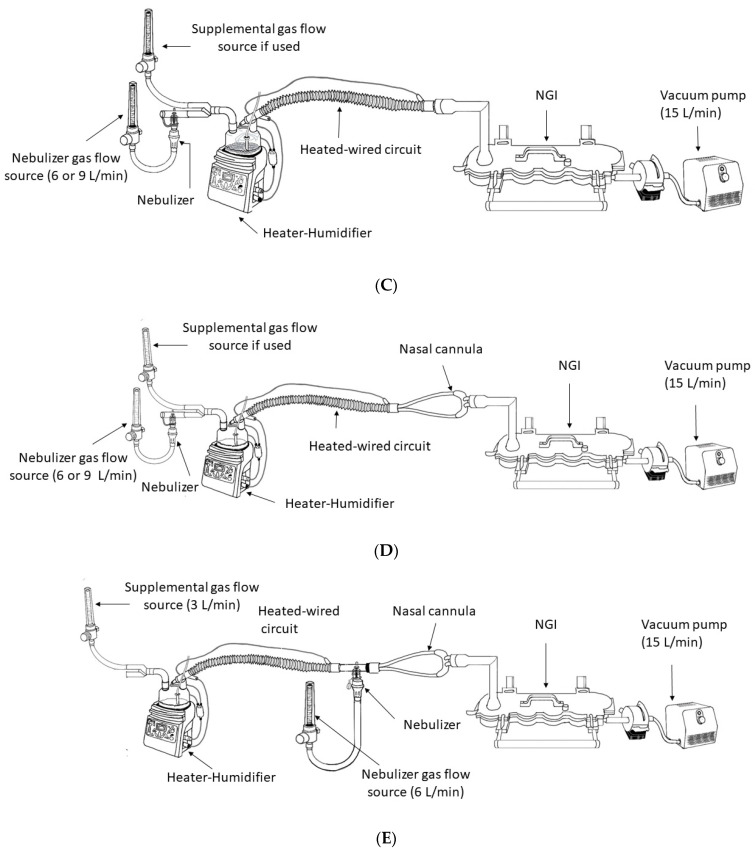
(**A**) Nebulizer with the Tee-piece connected to the induction port of the Next Generator Impactor (NGI). (**B**) Nebulizer with the Tee-piece connected to the Y-piece and connected to the induction port of the Next Generator Impactor (NGI). (**C**) Nebulizer with the Tee-piece connected to the Y-piece, connected to the humidifier inlet, connected to the heated-wired circuit and connected to the induction port of the Next Generator Impactor (NGI). (**D**) Nebulizer with Tee-piece connected to Y-piece, connected to the humidifier inlet, connected to the heated-wired circuit, connected to nasal cannula that was connected to the induction port of the Next Generator Impactor (NGI). (**E**) Nebulizer with Tee-piece placed between the heated-wired circuit and a large cannula that was connected to the induction port of the Next Generator Impactor (NGI).

Similarly to previous studies, the point of exit of the aerosol was connected to the IP of the NGI [4,5,6,7,8]. Twenty-seven different scenarios were tested to help identify the contribution of each component to the final aerosol characteristics (Table 3). First, the nebulizer connected to the Tee-piece was connected to the IP of the NGI (Figure 5A). Then, the nebulizer connected to the Tee-piece was connected to the Y-piece that was connected to the IP of the NGI (Figure 5B). Then, the nebulizer connected to the Tee-piece connected to the Y-piece was connected to the inlet of the humidifier that was connected to the circuit and connected to the IP of the NGI (Figure 5C). Then, small, large, and XXL cannulas were added to the system without additional flow and connected to the IP of the NGI (Figure 5D). Then, the tubing of the large cannula was disconnected from the circuit connector and connected to the IP of the NGI. The previous configurations were tested with the nebulizer operated at 6 and 9 L/min without additional flow. Additionally, the large and XXL cannulas were tested with additional flows of 3, 6, and 9 L/min provided through the Y-piece when the nebulizer was operated at 6 L/min, and 3 and 6 L/min when the nebulizer was operated at 9 L/min (Figure 5D). The nebulizer operated at 6 L/min with 3 L/min additional flow was tested with placement before the large cannula and connected to the IP of the NGI (Figure 5E). The large cannula was also tested with the nebulizer operated at 6 and 9 L/min and connected to the humidifier with the Tee-piece or with the Tee-piece connected to a Y-piece. All testing was conducted in triplicate.

The outcome measures were the mass median aerodynamic diameter (MMAD), geometric standard deviation (GSD), albuterol mass captured in the NGI from stage 1 to internal filter (MASS) fine particle fraction (mass of particles <5 µm in MASS/MASS *100) (FPF), and were calculated with CITDAS V3.1 software (Copley Scientific, Nottingham, UK). The results were expressed as mean ± SD with MMAD, FPF, and MASS reported in µg, percentage, and percentage of loading dose, respectively. We also compared the albuterol mass retained in the cannula. The solution output was calculated as the difference in nebulizer weight between the beginning and end of nebulization. The amount of albuterol remaining in the nebulizer cup was calculated as follows: albuterol concentration of nebulizer post treatment eluted with 10 mL of double distilled water times (post treatment with the addition of 10 mL of the double-distilled water nebulizer weight minus dry nebulizer weight). The solution output and amount of albuterol remaining in the nebulizer were used as the quality controls.

### 3.3. Statistical Analysis

A comparison of the outcomes at the same flow (6 or 9 L/min) among different configurations was conducted with analysis of variance followed by Dunnett’s test (nebulizer with the Tee-piece used as the control) and Tukey’s test for multiple comparisons. A comparison of the outcomes between the 6 and 9 L/min data was conducted with the unpaired T-test for unequal variances. A *p* value < 0.05 was considered statistically significant. A statistical software package was used for all of the calculations (Prism 9.4, GraphPad Software, La Jolla, CA, USA).

## 4. Conclusions

The delivery of aerosolized albuterol generated by a continuous output nebulizer placed in the inlet of a heated high flow nasal cannula system is feasible when large or XXL cannula sizes are used. The latter provided more efficient delivery without drug retention in the cannula. The MMAD and FPF of the aerosols make them suitable for intrapulmonary deposition. Adding supplemental flow significantly reduced the delivered MASS. The initial particle size selection occurred at the exit of the HFNC system circuit and the nasal cannula size further selected aerosols and reduced the drug delivery. The generating flow of the nebulizer did not affect the delivered MASS, but higher flows resulted in smaller particle size aerosols.

## Figures and Tables

**Table 1:** Outcome measures with the nebulizer operated at 6 and 9 L/min.

	Flow (L/min)	Neb Tee-	Neb Y	Neb Y + Circuit	Neb Y + Circuit + XXL	Neb Y + Circuit + L	Neb Y + Circuit + S
MMAD (µm)	6	4.55 ± 0.30 *	3.88 ± 0.12 *	2.39 ± 0.26 *	2.14 ± 0.21 *	1.86 ± 0.06 *	N/A
	9	3.46 ± 0.04	3.10 ± 0.10	1.90 ± 0.07	1.82 ± 0.07	1.59 ± 0.06	N/A
GSD	6	1.89 ± 0.11	1.85 ± 0.24	1.51 ± 0.09 *	1.45 ± 0.04 *	1.53 ± 0.23 *	N/A
	9	1.88 ± 0.09	1.83 ± 0.09	1.40 ± 0.02	1.35 ± 0.04	1.35 ± 0.02	N/A
FPF (%)	6	56.1 ± 4.4 *	65.44 ± 1.43 *	99.95 ± 0.01	99.96 ± 0.01	99.97 ± 0.01	100.00 ± 0.00
	9	71.04 ± 1.04	78.40 ± 3.72	99.97 ± 0.01	99.98 ± 0.01	99.88 ± 0.20	100.00 ± 0.00
MASS (% loading dose)	6	21.39 ± 2.88	20.47 ± 1.88	6.01 ± 0.59	7.50 ± 2.47	4.33 ± 1.55	0.10 ± 0.13
	9	21.38 ± 4.52	21.11 ± 3.46	9.12 ± 1.85	8.69 ± 0.30	3.97 ± 0.75	0.20 ± 0.10

MMAD = mass median aerodynamic diameter. GSD = geometric standard deviation. FPF = fine particle fraction. MASS = albuterol mass captured in the impactor from stage 1 to internal filter. Neb Tee = nebulizer with Tee-piece alone. Neb Y = nebulizer with Y-piece alone. Neb Y + Circuit = nebulizer placed in the inlet of the humidifier that was connected to the circuit. XXL = XXL cannula. L = large cannula. S = small cannula. * *p* values < 0.05 between 6 and 9 L/min.

**Table 2:** Outcome measures with the nebulizer operated at 6 and 9 L/min with additional flow from the heated flow nasal cannula system.

	Flow (L/min)	Large Cannula				XXL Cannula			
Additional Flow (L/min)		0	3	6	9	0	3	6	9
MMAD (µm)	6	1.86 ± 0.06	1.78 ± 0.06	N/A	N/A	2.14 ± 0.21	2.04 ± 0.18	2.04 ± 0.13	1.94 ± 0.13
	9	1.59 ± 0.06	1.64 ± 0.06	N/A	N/P	1.82 ± 0.07	1.69 ± 0.07	1.70 ± 0.05	N/P
GSD	6	1.53 ± 0.23	1.35 ± 0.10	N/A	N/A	1.45 ± 0.04	1.35 ± 0.07	1.33 ± 0.07	1.36 ± 0.11
	9	1.35 ± 0.02	1.39 ± 0.06	N/A	N/P	1.35 ± 0.04	130 ± 0.03	1.29 ± 0.03	N/P
FPF (%)	6	99.97 ± 0.01	95.78 ± 7.32	100.00 ± 0.00	100.00 ± 0.00	99.96 ± 0.01	99.97 ± 0.01	99.97 ± 0.01	99.40 ± 1.05
	9	99.88 ± 0.20	100.00 ± 0.00	100.00 ± 0.00	N/P	99.98 ± 0.20	100.00 ± 0.00	100.00 ± 0.00	N/P
MASS (% loading dose)	6	4.33 ± 1.55	2.59 ± 1.04	0.80 ± 0.60 *	0.25 ± 0.43 *	7.50 ± 2.47	6.20 ± 0.36 *	4.26 ± 1.14 *	3.04 ± 0.48 *
	9	3.97 ± 0.75	3.57 ± 0.65	0.37 ± 0.35 *	N/P	8.69 ± 0.30	4.57 ± 0.77 *	3.32 ± 0.75 *	N/P

MMAD = mass median aerodynamic diameter. GSD = geometric standard deviation. FPF = fine particle fraction. MASS = albuterol mass captured in the impactor from stage 1 to internal filter. N/A = not able to calculate. N/P = not performed. * *p* values < 0.05 between 0 and additional flow.

**Table 3:** Configurations of the tested scenarios.

	Nebulizer with Adapter			Position in the Circuit		Cannula			
Number	Tee-Piece Alone/Flow (L/min)	Tee-Piece Connected to Y-Piece/Flow (L/min)	Additional Flow (L/min)	Inlet of Humidifier	Before the Cannula	S	L	L-Tee-Piece ^#^	XXL
1	YES/6	NO	NO	NO	NO	NO	NO	NO	NO
2	NO	YES/6	NO	NO	NO	NO	NO	NO	NO
3	NO	YES/6	NO	YES	NO	NO	NO	NO	NO
4	NO	YES/6	NO	YES	NO	YES	NO	NO	NO
5	NO	YES/6	NO	YES	NO	NO	YES	NO	NO
6	NO	YES/6	NO	YES	NO	NO	YES *	NO	NO
7	NO	YES/6	NO	YES	NO	NO	NO	YES	NO
8	NO	YES/6	NO	YES	NO	NO	NO	NO	YES
9	NO	YES/6	3	YES	NO	NO	YES	NO	NO
10	NO	YES/6	3	YES	NO	NO	NO	NO	YES
11	NO	YES/6	3	NO	YES	NO	YES	NO	NO
12	NO	YES/6	6	YES	NO	NO	YES	NO	NO
13	NO	YES/6	6	YES	NO	NO	NO	NO	YES
14	NO	YES/6	9	YES	NO	NO	YES	NO	NO
15	NO	YES/6	9	YES	NO	NO	NO	NO	YES
16	YES/9	NO	NO	NO	NO	NO	NO	NO	NO
17	NO	YES/9	NO	NO	NO	NO	NO	NO	NO
18	NO	YES/9	NO	YES	NO	NO	NO	NO	NO
19	NO	YES/9	NO	YES	NO	YES	NO	NO	NO
20	NO	YES/9	NO	YES	NO	NO	YES	NO	NO
21	NO	YES/9	NO	YES	NO	NO	YES *	NO	NO
22	NO	YES/9	NO	YES	NO	NO	NO	YES	NO
23	NO	YES/9	NO	YES	NO	NO	NO	NO	YES
24	NO	YES/9	3	YES	NO	NO	YES	NO	NO
25	NO	YES/9	3	YES	NO	NO	NO	NO	YES
26	NO	YES/9	6	YES	NO	NO	YES	NO	NO
27	NO	YES/9	6	YES	NO	NO	NO	NO	YES

* Cannula disconnected from its adapter to the circuit. ^#^ Nebulizer with the Tee-piece connected to the inlet of the humidifier.

## Data Availability

Data are contained within the article.

## References

[1] Lee J.H., Rehder K.J., Williford L., Cheifetz I.M., Turner D.A. (2013). Use of high flow nasal cannula in critically ill infants, children, and adults: A critical review of the literature. Intensive Care Med..

[2] Gates R.M., Haynes K.E., Rehder K.J., Zimmerman K.O., Rotta A.T., Miller A.G. (2021). High-Flow Nasal Cannula in Pediatric Critical Asthma. Respir. Care.

[3] Miller A.G., Gentle M.A., Tyler L.M., Napolitano N. (2018). High-flow nasal cannula in pediatric patients: A survey of clinical practice. Respir. Care.

[4] Bhashyam A.R., Wolf M.T., Marcinkowski A.L., Saville A., Thomas K., Carcillo J.A., Corcoran T.E. (2008). Aerosol delivery through nasal cannulas: An in vitro study. J. Aerosol. Med. Pulm. Drug Deliv..

[5] Perry S.A., Kesser K.C., Geller D.E., Selhorst D.M., Rendle J.K., Hertzog J.H. (2013). Influences of cannula size and flow rate on aerosol drug delivery through the Vapotherm humidified high-flow nasal cannula system. Pediatr. Crit. Care Med..

[6] Watts A.B., Batra A.K., Tarbox T.N., Mc Williams B. (2015). Predication of aerool dose from a cannula using a bench-top infant simulated breathing model. Respir. Drug Deliv. Eur..

[7] Réminiac F., Vecellio L., Loughlin R.M., Le Pennec D., Cabrera M., Vourc’h N.H., Fink J.B., Ehrmann S. (2017). Nasal high flow nebulization in infants and toddlers: An in vitro and in vivo scintigraphic study. Pediatr. Pulmonol..

[8] Bennett G., Joyce M., Sweeney L., MacLoughlin R. (2018). In Vitro Determination of the Main Effects in the Design of High-Flow Nasal Therapy Systems with Respect to Aerosol Performance. Pulm. Ther..

[9] Berlinski A., Volsko T.E., Barnhart S.L. (2022). Aerosol therapy. Foundations in Neonatal and Respiratory Care.

[10] Li J., Gong L., Ari A., Fink J.B. (2019). Decrease the flow setting to improve trans-nasal pulmonary aerosol delivery via “high-flow nasal cannula” to infants and toddlers. Pediatr. Pulmonol..

[11] Réminiac F., Vecellio L., Heuzé-Vourc’h N., Petitcollin A., Respaud R., Cabrera M., Pennec D.L., Diot P., Ehrmann S. (2016). Aerosol Therapy in Adults Receiving High Flow Nasal Cannula Oxygen Therapy. J. Aerosol. Med. Pulm. Drug Deliv..

[12] Laube B.L., Janssens H.M., de Jongh F.H., Devadason S.G., Dhand R., Diot P., Everard M.L., Horvath I., Navalesi P., Voshaar T. (2011). What the pulmonary specialist should know about the new inhalation therapies. Eur. Respir. J..

[13] Schüepp K.G., Jauernig J., Janssens H.M., Tiddens H.A., Straub D.A., Stangl R., Keller M., Wildhaber J.H. (2005). In vitro determination of the optimal particle size for nebulized aerosol delivery to infants. J. Aerosol. Med..

[14] Berlinski A., Hayden J.B. (2010). Optimization of a procedure used to measure aerosol characteristics of nebulized solutions using a cooled next generation impactor. J. Aerosol. Med. Pulm. Drug Deliv..

[15] Marple V.A., Olson B.A., Santhanakrishnan K., Roberts D.L., Mitchell J.P., Hudson-Curtis B.L. (2004). Next generation pharmaceutical impactor: A new impactor for pharmaceutical inhaler testing. Part III. extension of archival calibration to 15 L/min. J. Aerosol. Med..

